# Altered Salience‐Default Mode Network Dynamics in Subclinical Depression: A Preclustering‐Based Co‐Activation Pattern Analysis

**DOI:** 10.1002/cns.70736

**Published:** 2026-02-04

**Authors:** Bo Zhang, Zhinan Yu, Feifan Yan, Yiwei Sun, Jiao Ye, Xiaoya Liu, Shouliang Qi, Xinhua Wei, Shuang Liu, Dong Ming

**Affiliations:** ^1^ Tianjin International Joint Research Center for Neural Engineering, Academy of Medical Engineering and Translational Medicine, Medical School, Tianjin University Tianjin China; ^2^ State Key Laboratory of Advanced Medical Materials and Devices Tianjin China; ^3^ Haihe Laboratory of Brain‐Computer Interaction and Human‐Machine Integration Tianjin China; ^4^ College of Medicine and Biological Information Engineering Northeastern University Shenyang China; ^5^ Department of Radiology, Guangzhou First People's Hospital, School of Medicine South China University of Technology Guangzhou China

**Keywords:** default mode network, functional magnetic resonance imaging, salience network, subclinical depression

## Abstract

**Background:**

Neuroimaging studies frequently report aberrant spontaneous brain activity and functional connectivity within core functional networks, including the default mode network (DMN), frontoparietal network (FPN), and salience network (SN) in subclinical depression (SD). However, the dynamic coordination among these networks remains poorly understood, impeding comprehensive elucidation of the underlying neuropathology of SD.

**Methods:**

Resting‐state functional magnetic resonance imaging (fMRI) data were collected from subjects with SD (*n* = 26) and healthy controls (HCs, *n* = 33). A preclustering‐based co‐activation pattern method was developed to investigate the dynamic patterns of network coordination. Finally, machine learning analysis was conducted to evaluate the potential of network dynamics for clinical diagnosis.

**Results:**

Subjects with SD exhibited decreased dwell time in the SN and increased transition frequency from the SN to DMN, which was positively correlated with depressive severity. Furthermore, an ensemble learning model based on SN‐DMN dynamic features achieved a classification accuracy of 96.44% in distinguishing SD from HC.

**Conclusion:**

These findings underscore the potential of altered SN‐DMN dynamics as candidates for future neuroimaging markers of SD and support a neurocognitive model whereby altered SN‐DMN dynamic coordination makes subjects with SD more prone to internal directed attention biases, thereby contributing to self‐related depressive symptoms like rumination.

## Graphical abstract

A preclustering‐based co‐activation pattern method was developed to investigate the network dynamics in subclinical depression (SD). SD exhibited decreased dwell time in the salience network (SN) and increased transition frequency from the SN to the default mode network (DMN). Altered SN‐DMN dynamics could distinguish SD from HC greatly.

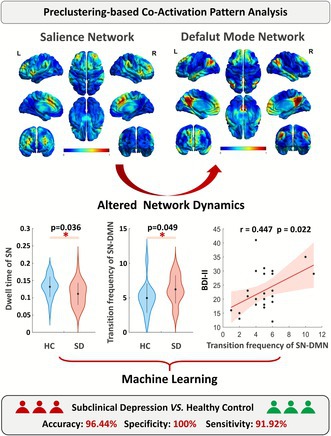

## 1 Introduction

Subclinical depression (SD), also known as subthreshold depression or subsyndromal depression, is a highly prevalent condition among a variety of populations. SD presents clinically significant symptoms of depression but does not meet the criteria for a positive diagnosis of major depressive disorder (MDD) (i.e., 2 to 5 symptoms of MDD according to the DSM‐V [1] and a score higher than 12 according to the Beck Depression Inventory‐II (BDI‐II), which could be seen as a precursor to depression [2, 3]). Although the symptoms presented in SD are not as severe as MDD, individuals with SD have frequently been reported to have deteriorated quality of life [4, 5] and increased suicide risks compared to non‐depressive subjects [6, 7]. Additionally, SD imposes a substantial burden on health services [8], given its high prevalence among the general population [9], up to 11.02% in a recent review from 113 studies [3]. However, the neuropathology of SD is largely unknown.

Functional magnetic resonance imaging (fMRI) studies have significantly advanced the understanding of the brain functional alterations underlying the neuropathology of SD. Emerging fMRI evidence indicates that SD is associated with abnormalities in large‐scale brain networks, particularly the default mode network (DMN), and the frontoparietal network (FPN) [10, 11, 12, 13, 14, 15, 16]. The DMN exhibits SD‐related alterations in spontaneous functional activity and functional connectivity, often linked to rumination (a negative and repetitive cognitive processing) or emotional‐related pathology [12, 13, 15, 17, 18]. Meanwhile, the FPN, also called executive control network shows decreased connectivity in SD and results in abnormalities in goal‐directed attention [10, 11, 14]. Notably, both DMN and FPN are regulated by the salience network (SN), which modulates the switch between internally directed cognition of the DMN and externally directed cognition of the FPN [19]. Altered functional coordination among DMN, SN, and FPN regions have been proposed to be associated with depressive symptoms like negative, repetitive introspection (i.e., rumination) [20] and attention biases towards negative, self‐referential information [21, 22]. Furthermore, a recent large‐scale study demonstrated that cortical expansion of the SN is a characteristic structural abnormality that is evident even in individuals before they meet full criteria for MDD and is present across a spectrum of depression severity [23], This suggests that SN expansion may represent an early neurodevelopmental risk marker. Therefore, it is plausible to hypothesize that the dysfunction of the DMN and FPN in SD may be rooted in abnormal cognitive modulation of the SN. This hypothesis requires further exploration of the relationships between brain networks from a more dynamic perspective.

Traditional resting‐state functional networks are typically estimated using the correlation coefficient among the time series of brain regions over extended periods of time [24]. However, this traditional approach is premised on the assumption that brain activity remains relatively static throughout an entire fMRI scan, overlooking the dynamic patterns of functional coordination as networks form and dissolve, as well as the patterns of transition between networks over time [25, 26]. Consequently, the dynamic transitions among SN, DMN, and FPN in subclinical depression, which involve modulation between internally and externally directed attention, are challenging to detect using traditional static functional network methods. Recently, co‐activation pattern (CAP) analysis has been introduced to track the dynamics of functional networks within individual time frames, providing a high‐resolution view of network dynamics up to a single time point of fMRI signals.

As a pivotal step in CAP analysis, brain activation determination plays a crucial role in eliminating redundant inactive information, enhancing clustering effects, and reducing computational complexity [27]. Brain activation determination involves identifying activated time points in the original Blood Oxygen Level Dependent (BOLD) time series of each voxel and excluding BOLD information from inactive time points. Existing studies typically set a specific activation ratio threshold (retaining signal intensities that exceed a predefined threshold), yet there is a lack of consensus regarding this activation ratio. Liu and Duyn first proposed a 15% activation ratio based on the similarity between CAP patterns and the default mode network. This activation ratio was widely adopted by later studies, despite the influence of collecting environment and demographic factors [25, 28]. Chen et al. employed a sliding window strategy to select a 31% activation ratio grounded in the variability of global Pearson correlations with respect to two resting‐state functional networks [27]. Kaiser et al. proposed an activation ratio determination strategy based on the mean intensity of whole‐brain BOLD signals [22]. The methodological differences directly affect the accuracy of brain network recognition and may contribute to the current inconsistency in identifying brain networks across CAP studies. To address this, this study proposed a preclustering‐based brain activation determination method. This method initially employs cluster analysis to identify the predominant activation state of each voxel, using the average signal intensity within this state as the baseline for determining brain activation. In contrast to traditional methods, this approach is independent of prior assumptions, data‐driven, and thus resilient to potential interference from data collection conditions (equipment, environment) or specific demographic factors (race, gender, age).

In this study, we propose a preclustering‐based co‐activation pattern (PC‐CAP) method to evaluate SD‐related alterations in brain network dynamics. Furthermore, we delved into the associations between metrics of network dynamics and depressive severity. Finally, a machine learning analysis was conducted to examine the reliability of our findings and the potential validity of functional network dynamic differences in the future diagnosis of SD.

## 2 Methods

### 2.1 Participants

Participants were recruited from 1105 college students who had undergone health screening at Guangzhou Medical University. All participants completed the Beck Depression Inventory II (BDI‐II) to assess depressive symptoms. A total of 34 SD individuals (11 males and 23 females) with BDI‐II scores exceeding 13 were included in this study. Concurrently, 40 individuals (21 males and 19 females) with BDI‐II scores below 5 were randomly selected from the same cohort to serve as the healthy control (HC) group. The HC group was matched with the SD group for age, sex, and educational background, and none of the controls exhibited any clinical depressive symptoms upon structured clinical assessment. Refer to Supporting Information or a previous publication for detailed information on inclusion criteria [29].

This study was approved by the Medical Ethics Committee of Tianjin University (Approval ID: TJUE‐2025‐323). All participants provided written informed consent after a detailed description of the study was provided.

### 2.2 Image Acquisition

A 3‐Tesla MRI scanner (Siemens, Erlangen, Germany) equipped with an 8‐channel phase‐array brain coil was used to obtain MRI images. During the acquisition process, foam pads and earphones were provided for the subjects to minimize head movement and reduce MRI noise.

Resting‐state scans were acquired using an echo planar imaging sequence with the following parameters: TE = 21 ms, TR = 2500 ms, FOV = 200 × 200 mm [2], flip angle = 90°, matrix = 64 × 64, voxel size = 3.5 × 3.1 × 3.1 mm^3^, timepoints = 200, and 40 slices with no gap. T1‐W images were acquired using a magnetization prepared rapid acquisition gradient echo sequence with the following parameters: TR = 2530 ms, TE = 2.34 ms, FA = 7°, FOV = 256 × 224 mm^2^, slice thickness = 1.0 mm with no gap.

Participants were instructed to keep their eyes closed, remain awake and relax without actively thinking during the scanning. All images were examined by two experienced radiologists to ensure that there were no lesions or artifacts. Participants and MRI scanning parameters were in line with our previous publications [17].

### 2.3 Imaging Preprocessing

Resting‐state fMRI data preprocessing was performed using the Data Processing Assistant for Resting‐State fMRI (DPARSF, http://www.restfmri.net) [30] on the MATLAB platform (The MathWorks, Natick). For each subject, the first 10 volumes were discarded. Images then underwent slice timing correction, head motion correction, normalization, nuisance covariates regression, bandpass filtering (0.01–0.08 Hz), and spatial smoothing. Refer to Supporting Information or a previous publication for detailed information on preprocessing [29].

For each participant, BOLD time series of preprocessed resting‐state fMRI data were extracted based on the Dosenbach functional atlas, which parcellates the whole brain into 160 functionally defined regions of interest (ROIs) [31]. Then, each time series was normalized using a z‐score to represent the relative activation intensity changes within each ROI. BOLD data of all subjects were concatenated into a matrix [(190 TRs × 59 subjects) × 160ROIs].

### 2.4 PC‐CAP Analysis

**FIGURE 1:**
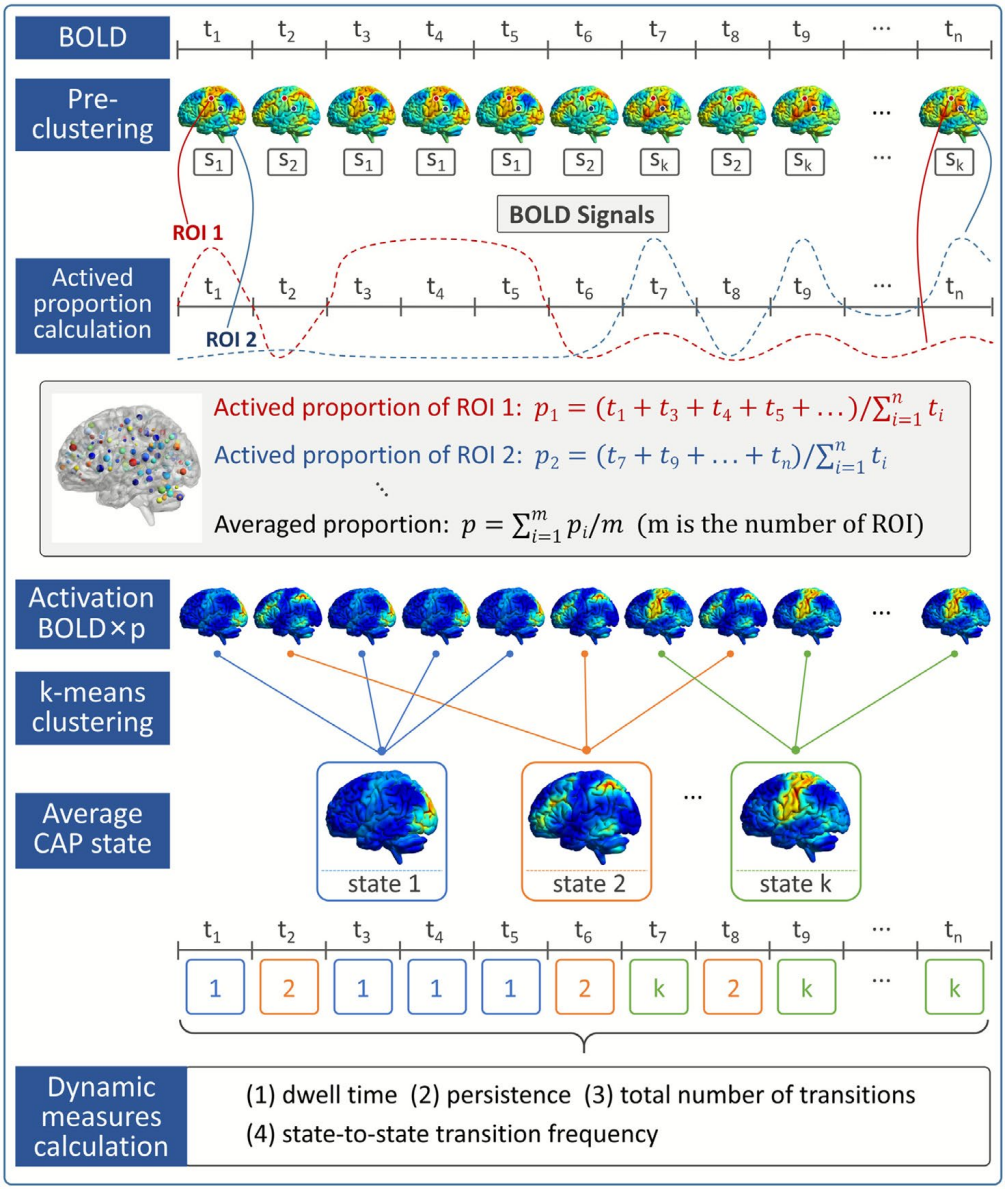
An illustration for the PC‐CAP analysis. First, preclustering‐based brain activation determination was performed on the initial time series to calculate the activation proportion. Then, activated BOLD time series of all subjects were concatenated and input for the *k*‐means clustering to partition the data into k brain states. Finally, dynamic measures of brain networks were calculated based on the brain state series.

PC‐CAP analysis is a data‐driven analytical technique that identifies recurring brain states with similar co‐activation patterns across the entire brain. In this study, PC‐CAP analysis was conducted using custom‐made scripts in MATLAB. The flowchart illustrating the PC‐CAP analysis process is shown in Figure 1.

A preclustering‐based brain activation determination was first conducted. Initially, *k*‐means clustering analysis was performed on the concatenated time series to identify *k* recurring patterns of co‐activation that emerge across participants and time points, along with the brain co‐activation states at each specific time point. This process, termed preclustering, involves the *k*‐means clustering process, where the first step consists of setting the number of clusters, denoted as *k*. Following strategies commonly adopted in previous studies, this research set *k* to range from 2 to 20 in increments of 1 [27, 32, 33]. The optimal value of *k* was determined using the elbow criterion and silhouette scores.

Subsequently, for each ROI *i*, the average BOLD signal intensities (*V*_1_, *V*_2_, *V*_3_, … *V*_*k*_) corresponding to each activation state (*S*_1_, *S*_2_, *S*_3_, … *S*_*k*_) were computed. The activation state with the highest average signal intensity was designated as the predominant activation state (*S*_*p*_) specific to ROI *i* (e.g., the *S*_*p*_ of the posterior cingulate cortex corresponds to the default mode network, while the *S*_*p*_ of the occipital lobe represents the visual network). Following this, the proportion of time points where the BOLD signal intensity surpassed the average intensity of the predominant state (*V*_*p*_) was calculated, representing the activation proportion (*p*_*i*_) of region i. This process was iterated across all 160 brain regions, yielding the activation proportion for each ROI. Subsequently, the mean activation proportion across all ROIs was computed and denoted as the final activation proportion (*p*). Lastly, within the *z*‐scored time series of each participant, data corresponding to the top p% of signal intensities were retained to signify brain activation, while the remaining signals were set to 0, thus completing the comprehensive process of brain activation determination.

Then, *k*‐means clustering analysis was performed based on the concatenated BOLD time series following activation determination, with *k* ranging from 2 to 20 in increments of 1. Both the elbow criterion and silhouette scores were utilized to ascertain the optimal clustering solution for *k*. Subsequently, the volumes assigned to each brain state were averaged and normalized by the within‐cluster standard deviation to produce CAP maps (Z‐maps) for further visualization and the identification of functional brain networks.

### 2.5 Brain Network Dynamic Measures

Four metrics were calculated to evaluate the dynamic properties of resting‐state brain networks: (1) Dwell time, representing the total proportion of volumes that the participant spent in that brain network state over the scan series; (2) Persistence, quantifying the average duration of consecutive timepoints a participant remains in a particular brain network state before transitioning to another state; (3) Total number of transitions, counting the switches between different brain network states across the scan series; (4) State‐to‐state transition frequency, measuring the frequency of transitions from one volume belonging to brain network state A to the next volume belonging to B. Dwell time and persistence reflect network state dominance, while the total number of transitions and state‐to‐state transition frequency characterize network dynamic transitions.

### 2.6 Statistical Analysis

Demographic data of participants were analyzed using the Statistical Package for the Social Sciences software (SPSS), version 17 (Chicago, IL). Two‐sample *t*‐tests were conducted to evaluate differences in age and education, and a chi‐squared test was performed to assess gender differences.

To explore the alterations of network dynamic properties in SD, dwell time, persistence, total number of transitions, and frequency of state‐to‐state transitions were compared between SD and HC groups. Initial comparisons were conducted using two‐sample *t*‐tests, and false discovery rate (FDR) corrections were applied for *t*‐tests. Furthermore, to control for the potential confounding effects of age and sex, metrics with significant differences in *t*‐tests were subsequently re‐analyzed using a General Linear Model (GLM) with group as the fixed factor and age and sex as covariates. Furthermore, to investigate the association between depressive severity and network dynamic properties, we performed partial correlation analysis (controlling for age and sex) between dynamic properties and BDI‐II scores in the SD group. All statistical and correlational analyses of network dynamic measures were performed using MATLAB.

### 2.7 Machine Learning Analysis

A machine learning classification analysis was conducted to further verify the reliability of the findings and explore the discriminating power of the network dynamic measures. We integrated features of dynamic measures with significant group differences. Subsequently, the data were normalized to a standardized range of 0 to 1. To enhance classification performance, we adopted an ensemble learning strategy utilizing support vector machines (SVMs) with bagging and parameter optimization. For the optimal configuration of SVM parameters, we executed a thorough cross‐validation process, exploring diverse permutations of the penalty coefficient (C) and kernel function hyperparameter (gamma). For model training, we implemented a bagging framework, which involved the generation of multiple training subsets through random resampling. These subsets were then leveraged to train an ensemble of SVM models, each utilizing distinct parameter combinations. The performance of each individual model was rigorously evaluated using a leave‐one‐out cross‐validation (LOOCV) scheme. For each test sample, predictions were made using an ensemble of SVM models. The final prediction was determined through a weighted voting mechanism, with the weights assigned to each model based on their respective training accuracies. To assess the performance of our ensemble learning model, we calculated the average accuracy, sensitivity, and specificity across ten iterations of cross‐validation.

## 3 Results

### 3.1 Demographic Data Comparisons

There were no significant differences in gender, age, or years of education between SD and HC subjects. The BDI‐II scores of SD subjects were significantly higher than those of HC subjects (*p* < 0.05). Detailed demographic and clinical data of participants are shown in Table 1.

**TABLE 1:** Demographic characteristics of the sample.

Characteristics	SD (*n* = 26)	HCs (*n* = 33)	*p*
Gender (male/female)	10/16	16/17	0.152^a^
Age (years)	19.69 ± 1.73	19.18 ± 0.87	0.593^b^
Education (years)	13.36 ± 0.92	13.18 ± 0.87	0.492^b^
BDI‐II scores	22.08 ± 7.34	1.76 ± 1.79	0.000^b^

*Note:* ^a^ and ^b^ indicate the *p* value for the Chi‐Square test and two‐sample *t*‐test respectively.

Abbreviations: BDI‐II, Beck Depression Inventory‐II; HCs, healthy controls; SD, subclinical depression.

### 3.2 Preclustering‐Based Brain Activation Determination

**FIGURE 2:**
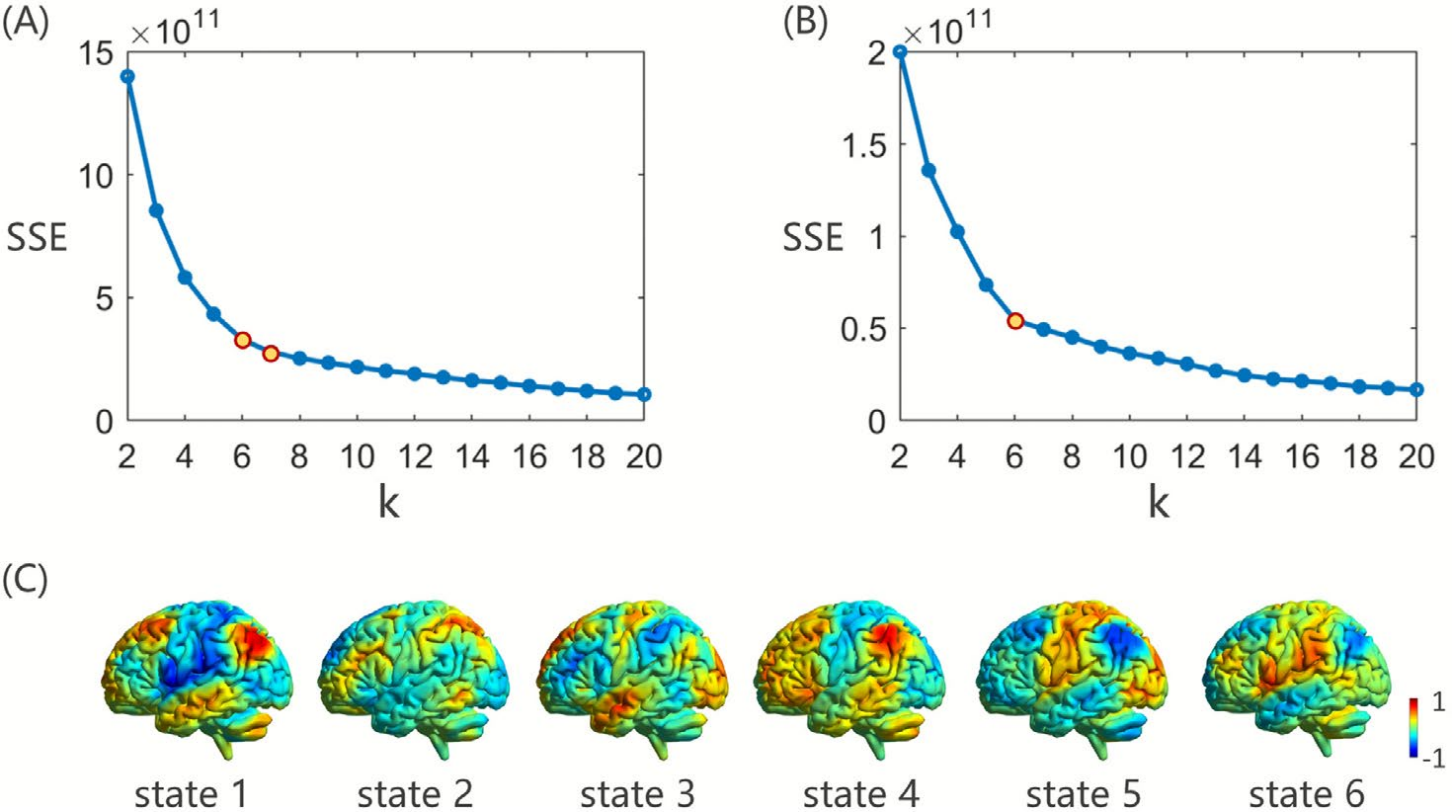
Selection of the optimal number of clusters. (A) The trend of the SSE with cluster numbers k in the preclustering process. (B) The trend of the sum of SSE with cluster numbers k in the clustering process after activation determination. (C) Six states derived from the preclustering process. SSE, sum of squared errors.

In the process of preclustering‐based brain activation determination, we initially utilized the elbow method to select the optimal number of clusters. The trend of the sum of squared errors (SSE) with respect to the number of clusters (k) is illustrated in Figure 2A. Although a distinct inflection point is not immediately apparent in the graph, a relatively pronounced change in the rate of variation emerges around *k* = 6 or 7, suggesting a relatively favorable clustering effect. Further evaluation using the silhouette coefficient method revealed that for *k* = 6, the average silhouette coefficient across all sample points was 0.058 (SD = 0.054), whereas for *k* = 7, it was slightly lower at 0.055 (SD = 0.051). It is important to note that a higher average silhouette coefficient signifies stronger intra‐cluster cohesion and clearer inter‐cluster separation, suggesting a superior clustering effect. Consequently, we selected *k* = 6 as the optimal cluster number. Six co‐activation patterns are presented in Figure 2C.

The predominant activation states of each of the 160 brain regions were analyzed. Based on the average BOLD signal intensity under the predominant activation states within each ROI, the activation proportion was further calculated. The mean activation proportion across all ROIs was determined to be 23% (SD = 0.063), which represents the whole‐brain activation ratio. Furthermore, the study conducted a comparative analysis of the activation proportions between subjects with SD and HCs, and no significant difference was found between the groups. Consequently, a 23% threshold was employed for brain activation determination for each participant. BOLD data corresponding to the top 23% of signal intensities were retained and designated as brain activation, whereas the remaining inactive signals were set to zero. Subsequently, the time series from all participants were concatenated along the temporal dimension for further cluster analysis.

### 3.3 Recurrent Brain Network States

**FIGURE 3:**
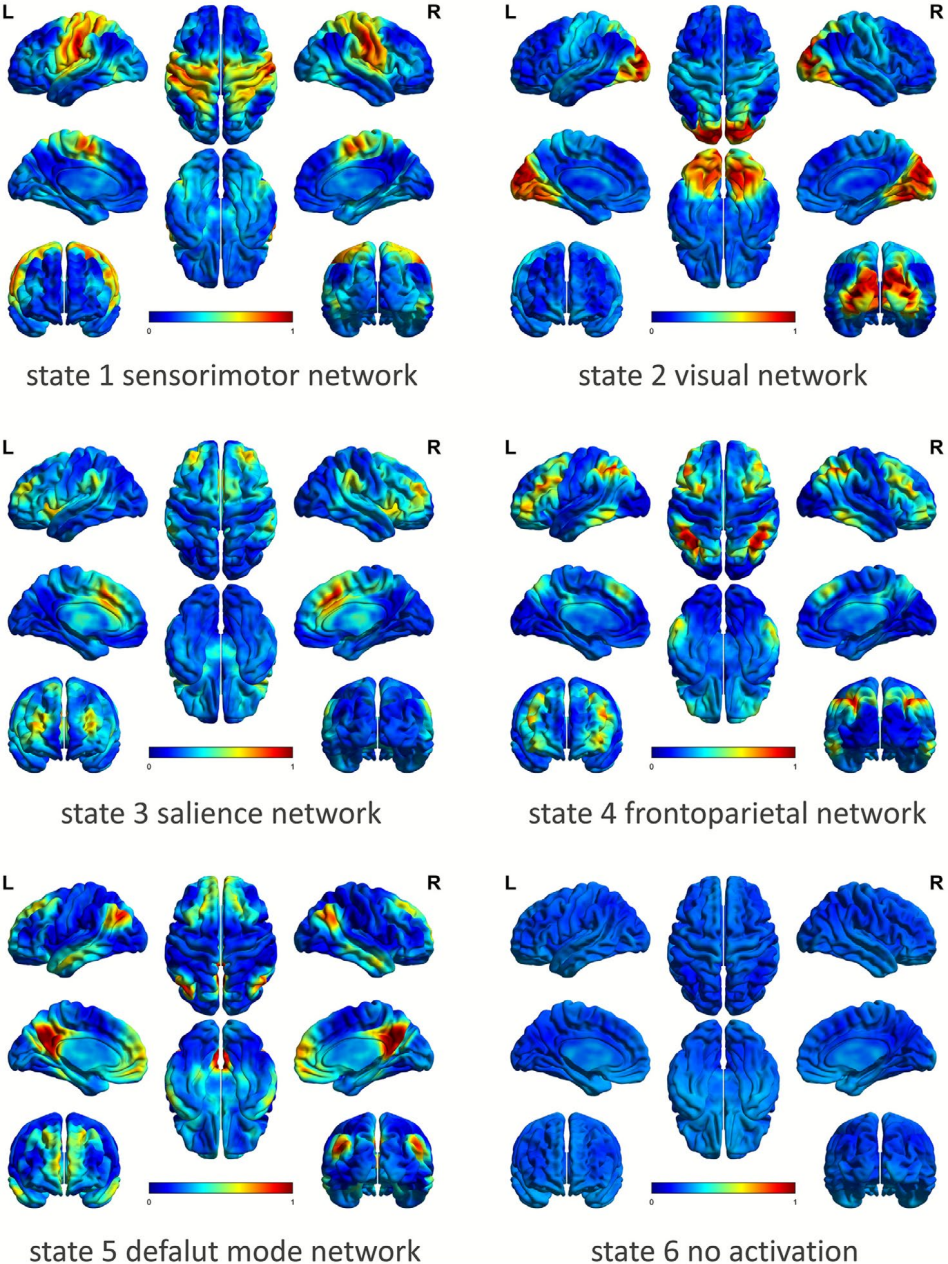
Five PC‐CAP states derived from k‐means clustering. These brain states were normalized at the group level. The red color represents a relatively stronger activation, while the blue color indicates a relatively stronger deactivation. State 1 was identified as sensorimotor network with the co‐activation of the precentral gyrus and postcentral gyrus. State 2 represented visual network with the broad co‐activation of the lateral and medial occipital lobe. State 3 was identified as salience (or ventral attention) network characterized by the co‐activation of anterior cingulate cortex and anterior insula. State 4 was identified as frontoparietal network which included the dorsolateral prefrontal lobe, lateral parietal cortex, middle and inferior temporal gyri. State 5 was identified as default mode network with the co‐activation of the posterior cingulate cortex, precuneus, angular gyrus, and medial prefrontal cortex. State 6 was identified as an inactive state.

*K*‐means clustering analysis was performed based on the concatenated BOLD time series after activation determination. The elbow criterion was applied to identify the optimal clustering solution of *k* = 6, as shown in Figure 2B. When *k* = 6, the line chart exhibits the maximum distortion, signifying the most favorable clustering outcome and highlighting enhanced clustering effectiveness compared to preclustering. Six co‐activation patterns were further visualized in Figure 3, with five patterns exhibiting significant co‐activation across multiple brain regions, while the remaining pattern represents an inactive brain state.

As shown in Figure 3, state 1 indicates the co‐activation of the precentral gyrus and postcentral gyri, identified as the sensorimotor network (SMN), implicated in motor processes and somatosensation. State 2 includes the co‐activation of the lateral and medial occipital lobe broadly, clearly representing a visual network (VN). State 3 is characterized by the co‐activation of the anterior cingulate cortex (ACC) and anterior insula, aligning closely with the SN. State 4 comprises the dorsolateral prefrontal lobe (DLPFC), lateral parietal cortex, middle and inferior temporal gyri, identified as the FPN. State 5 is characterized by the co‐activation of the posterior cingulate cortex, precuneus, angular gyrus, and medial prefrontal cortex (MPFC), which aligns well with regions of the DMN. State 6 indicates an inactive brain state.

### 3.4 SD‐Related Alterations in Network Dynamic Measures

**FIGURE 4:**
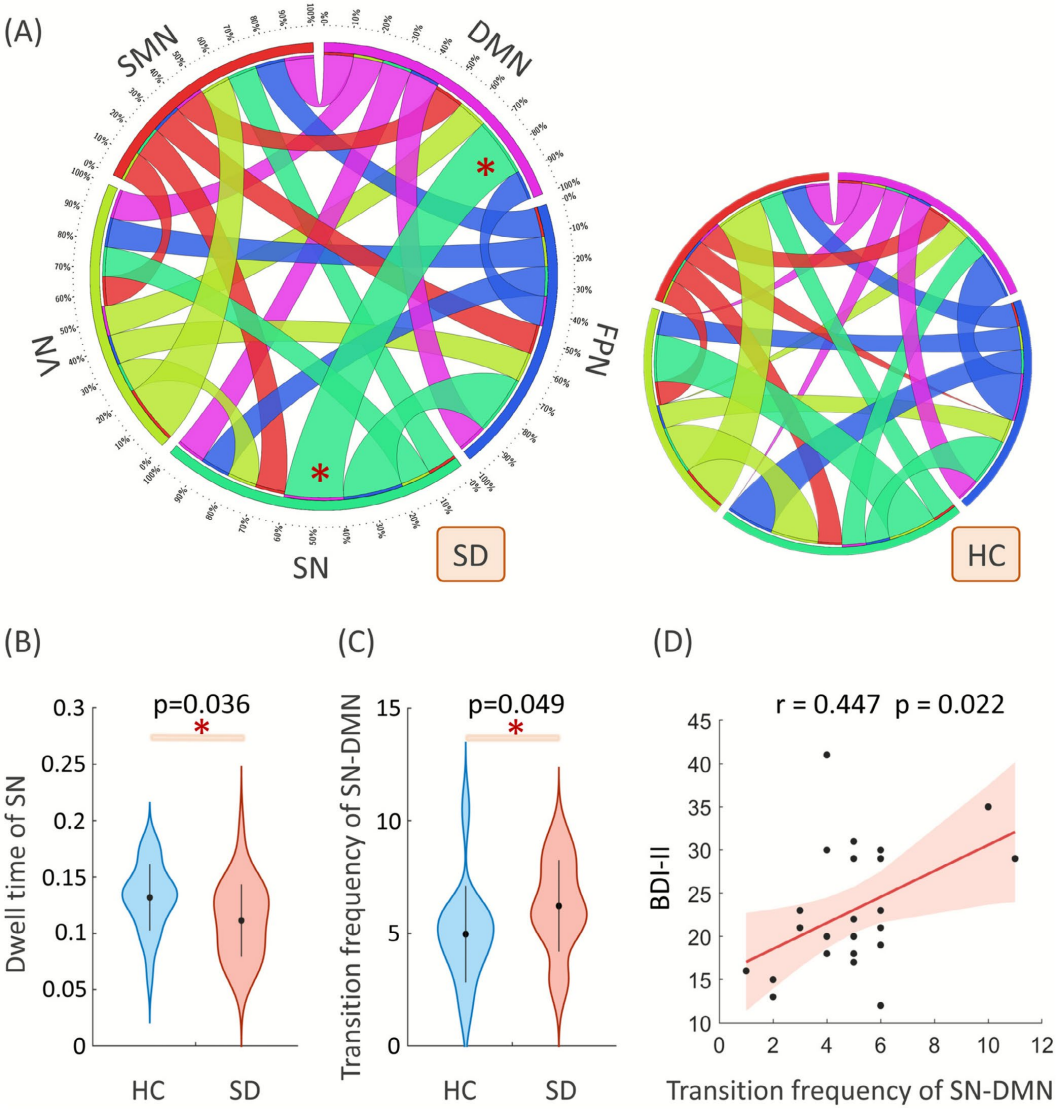
Network dynamic alterations in SD. (A) the visualization of state‐to‐state transition frequency in SD and HC groups. The red, yellow, green, blue, and purple colors represent the transition starts from SMN, VN, SN, FPN, and DMN respectively. The thick line represents a relatively higher transition frequency and vice versa. (B) The dwell time of the SN in subjects with SD and HC. Plots represent the distribution of data for each group, along with each subject's data points. (C) The transition frequency from SN to DMN in subjects with SD and HC. (D) The correlation between the transition frequency from SN to DMN and BDI‐II scores in SD group. BDI‐II, Beck Depression Inventory‐II; DMN, default mode network; FPN, frontoparietal network; HC, healthy control; SD, subclinical depression; SMN, sensorimotor network; SN, salience network; VN, visual network; *p < 0.05.

Dwell time, persistence, total number of transitions, and state‐to‐state transition frequency were compared between SD and HC groups. The results of two‐sample *t*‐test showed that the dwell time of SN was significantly decreased in subjects with SD (*p* = 0.014, FDR corrected). Network transition frequency from SN to DMN was significantly increased in subjects with SD (*p* = 0.026, FDR corrected), but no difference was found between SN and FPN, or between DMN and FPN. There was no alteration in the persistence of any CAP state and total number of transitions. Furthermore, to rigorously control for the potential influence of age and sex, these group differences were further validated using GLM analyses. After controlling for these covariates, the SD group continued to demonstrate a significantly decreased dwell time in the SN compared to the HC group (*β* = −0.0176, 95% CI [−0.0340, −0.0012], *p* = 0.036; Cohen's *d* = −0.67), as shown in Figure 4B. Conversely, the transition frequency from SN to DMN remained significantly increased in the SD group (*β* = 1.124, 95% CI [0.0021, 2.2462], *p* = 0.049; Cohen's *d* = 0.60), as shown in Figure 4A,C. The effects of sex and age were not significant in either model.

In addition, to further investigate the association between depressive severity and network dynamic properties, partial correlation analysis was performed and showed that network transition frequency from SN to DMN was positively correlated with BDI‐II scores (*r* = 0.447, *p* = 0.022), as shown in Figure 4D.

### 3.5 Classification Performances of Ensemble Learning

The dwell time features of the SN and the transition frequency from SN to the DMN were extracted for each individual. Utilizing these network dynamic features, an ensemble learning model employing SVM classifiers with a bagging approach achieved a classification accuracy of 96.44% ± 2.03% in distinguishing individuals with SD from HC, with 100% ± 0% specificity, 91.92% ± 4.60% sensitivity, highlighting its effectiveness in accurately identifying individuals with SD.

## 4 Discussion

The current study conducted a PC‐CAP analysis to evaluate SD‐related alterations in brain network dynamics. By comparing dynamic dominance and transition measures, the current study provided novel evidence that subjects with SD exhibited a decreased dwell time in the SN. Furthermore, the network transition frequency from the SN to DMN was significantly increased in subjects with SD, and this increase was positively correlated with depressive severity. These findings offer fresh insights into the alterations of brain functional dynamics in SD, facilitating the comprehensive understanding of the brain dysfunction and neuropathology underlying SD.

Based on the novel PC‐CAP method, this study accurately identified five functional networks with recurring patterns of co‐activation, including SMN, VN, SN, DMN, and FPN. Compared to brain activation determination methods based on prior knowledge in previous studies [25, 27, 28, 34], the PC‐CAP method could accurately identify the most significantly activated network at each time point. This method effectively circumvents the issue of identifying ambiguous states where multiple networks are simultaneously activated and potentially interfering with each other. This is particularly advantageous for exploring the information communication or causal relationships among the DMN, SN, and FPN networks in this study. In addition, the PC‐CAP method operates independently of prior assumptions, rendering it resilient to potential biases stemming from data collection conditions or specific demographic factors (race, gender, age). Therefore, the PC‐CAP method could provide methodological guidance for the future analysis of brain function dynamics.

Previous static fMRI studies have robustly demonstrated that dysfunction of the DMN is associated with the neuropathology of depression [35, 36, 37, 38]. In MDD, several recent meta‐analyses of static functional connectivity studies, leveraging multi‐center data and large sample sizes, have consistently shown increased strength and decreased stability of functional connectivity within key DMN regions in patients with MDD [36, 37, 39]. Similarly, in SD, previous studies examining the amplitude of low‐frequency fluctuations and regional homogeneity have revealed widespread alterations in spontaneous neural activities within DMN regions, including the posterior cingulate cortex, precuneus, hippocampus, anterior cingulate cortex (ACC), and medial prefrontal cortex [17, 18, 40]. According to FC studies, Li et al. reported altered FC patterns in the ACC of elderly individuals with SD [41], while Philippi et al. further demonstrated that the severity of SD was associated with distinct FC patterns in the ACC, a pivotal region within the DMN [42]. The DMN is thought to mediate psychological processes such as introspection and mental movement away from externally concentrated thoughts. Dysfunction of the DMN has been demonstrated to be positively correlated with rumination [43], which acts as remarkable clinical manifestations of depressive patients. Therefore, static dysfunction of the DMN was recognized as a characteristic of brain impairment and a key structure in understanding the neuropathology underlying both MDD and SD.

However, the present findings indicated that the DMN dysfunction may not be the source of functional alterations in depression, and greater attention should be paid to the SN and its transitions with the DMN. It is well‐established that the SN modulates the switch between internally directed cognition mediated by the DMN and externally directed cognition facilitated by the FPN [19]. Consequently, the present study closely examined the dynamic relationship among SD, DMN, and FPN. The results provided evidence that the transition frequency from SN to DMN was significantly higher in subjects with SD than HC, and it was positively correlated with the severity of depression. These findings indicate that dynamic coordination between SN and DMN systems may play a role in directing attention towards internal thoughts, making these regions particularly pertinent to biases towards rumination [19, 21, 44]. Moreover, these dynamic alterations may be overlooked in static studies based on larger time scales, resulting in reduced attention being paid to the role of abnormal attention regulation mechanism in SD. Consequently, the static dysfunction of the DMN observed in SD might merely be superficial, while the heightened transition frequency from the SN to the DMN could be the underlying cause for the intensified functional activity and connectivity within the DMN. This supports a neurocognitive model in which abnormal attention biases towards introspection and self‐referential information make SD subjects more prone to rumination. Additionally, it should also be noted that regarding the FPN, we did not observe altered dynamic patterns in SD that are analogous to those seen in MDD [22]. This result may suggest that the regulation of goal‐directed attention towards the external world remains unaffected in SD, potentially enhancing the ability to disengage from rumination and mitigating the development of depression from subclinical stage to major depression.

To further validate the reliability of our findings and the discriminative ability of network dynamics in clinical diagnosis, an ensemble learning model utilizing these network dynamics features achieved a classification accuracy of 96.44% in distinguishing individuals with SD from HC. This indicates that network dynamics related to the DMN and SN might provide candidates for future neuroimaging markers of SD for clinical diagnosis. Previous research has employed various feature modalities, such as combined brain structural and functional features [10, 45], spontaneous brain functional activity features [18], connectivity features involving the lateral habenula and thalamus [46], whole‐brain functional connectivity features [47], task‐based brain activation during emotional processing features [48], and EEG spectral and connectivity features [49], achieving SD identification accuracies ranging from 77.97% to a peak of 92%. In comparison, our study achieved superior classification performance with the lowest feature dimensionality, which was attributed to the high specificity of the SN‐DMN dynamics features and the effectiveness of the ensemble learning algorithm. These further underscore the significant role of SN‐DMN dynamics in elucidating the neuropathology of SD.

It is noteworthy that these findings hold potential clinical translational implications for the selection of repetitive transcranial magnetic stimulation (rTMS) treatment targets. rTMS, a non‐invasive and promising therapy for depression, has garnered approval from the U.S. Food and Drug Administration (FDA). The DLPFC is the most widely stimulated site for depression, with its efficacy confirmed by numerous previous studies [50, 51]. The findings of this study suggest that the neural basis of DLPFC treatment's effectiveness may reside in its crucial connection with the ACC, which is a core brain region of the SN. The stimulation of the DLPFC can facilitate the transition of brain states from the SN to FPN while inhibiting the transition from the SN to DMN. This, in turn, suppresses excessive self‐referential attention related to rumination and may constitute an essential neurobiological basis for rTMS treatment targeting the DLPFC.

Several limitations should be mentioned. Firstly, the scanning time of resting‐state fMRI in the current study was approximately 8 min (200 time points), resulting in a relatively limited number of frames for PC‐CAP analysis. This limitation inevitably impacted the robustness and reliability of the dynamic properties examined. Future endeavors could enhance these aspects by extending the scan duration and advancing the time resolution of imaging techniques. Secondly, while this study utilized PC‐CAP analysis to investigate dynamic transitions between co‐activation states (large‐scale networks), the neural mechanisms underlying these network dynamics remain elusive [52]. A promising approach to uncovering more precise dynamic information on a finer temporal scale and gaining deeper insights into both static and dynamic neural mechanisms is through the integration of fMRI and EEG technologies [53]. Third, although the SD subjects were recruited and screened from a pool of over 1000 college students, the sample size remained relatively modest, potentially limiting the statistical strength of our findings. Consequently, future studies are encouraged to employ multiple‐cohort data with larger sample sizes to bolster the generalizability and statistical power of their results.

## 5 Conclusion

In conclusion, the present study utilized PC‐CAP analysis to identify brain networks characterized by recurring spatial co‐activation states over time and compared dynamic measures of these networks between subjects with SD and HCs. Our results offer fresh evidence that individuals with SD exhibit decreased dwell time in the SN and increased transition frequency from the SN to the DMN. An ensemble learning model, based on these SN‐DMN dynamic features, achieved a classification accuracy of 96.44% in distinguishing SD from HC. These findings underscore the potential of altered SN‐DMN dynamics as candidates for future neuroimaging markers of SD and lend support to a neurocognitive model whereby altered SN‐DMN dynamic coordination makes subjects with SD more prone to internal directed attention biases, thereby contributing to self‐related depressive symptoms like rumination.

## Funding

This work was supported by the National Key Research and Development Program of China (Grant 2023YFF1203700), the Natural Science Foundation of Tianjin (Grant 24ZXZSSS00330), the National Natural Science Foundation of China (Grant 62376187), and the Autonomous Project of Haihe Laboratory of Brain‐Computer Interaction and Human‐Machine Integration (Grant 24HHNJSS00016).

## Ethics Statement

This study was approved by the Medical Ethics Committee of Tianjin University (Approval ID: TJUE‐2025‐323). All participants provided written informed consent after a detailed description of the study was provided.

## Conflicts of Interest

The authors declare no conflicts of interest.

## Supporting information


**Data S1:** cns70736‐sup‐0001‐Supinfo.docx.

## Data Availability

The data that support the findings of this study are available from the corresponding author upon reasonable request.
