# Supplementary material for: Altered Salience‐Default Mode Network Dynamics in Subclinical Depression: A Preclustering‐Based Co‐Activation Pattern Analysis
Source: CNS Neurosci Ther. 2026 Feb 4;32(2):e70736. doi: 10.1002/cns.70736 (PMC12871089; doi:10.1002/cns.70736)
Supplement: Supplementary file 1 — Data S1: cns70736‐sup‐0001‐Supinfo.docx. [file CNS-32-e70736-s001.docx]

#### Supplemental materials

## Detailed information on inclusion criteria

Participants were recruited from 1105 college students who had undergone health screening at Guangzhou Medical University. All participants completed the Beck Depression Inventory II (BDI-II) to assess depressive symptoms. The BDI-II consists of 21 items rated on a 4-point scale, with total scores ranging from 0 to 63. Among the 1105 students, individuals with BDI-II scores exceeding 13 were invited for a subsequent structured clinical interview based on the DSM-V. Individuals who exhibited core depressive symptoms but did not meet the diagnostic criteria for depression or any other psychiatric disorders were classified as having SD. Specifically, they showed at least one core depressive symptom and one occasional secondary symptom, but the total number of symptoms did not exceed four. A total of 34 SD individuals (11 males and 23 females) were included in this study. Concurrently, 40 individuals (21 males and 19 females) with BDI-II scores below 5 were randomly selected from the same cohort to serve as the healthy control (HC) group. This cut-off was chosen to ensure that the control group did not exhibit any depressive symptoms and was in a mentally healthy state. The HC group was matched with the SD group for age, sex, and educational background, and none of the controls exhibited any clinical depressive symptoms upon structured clinical assessment. None of the participants in either group met the diagnostic criteria for MDD or any other psychiatric disorders based on the DSM-V. The structural clinical interviews based on the DSM-V were conducted by two experienced psychiatrists at Guangzhou First People's Hospital.

Other inclusion criteria for all participants were an age range of 19 to 25 years, right-handedness, no visible lesions on MRI scans, no neurological disorders, no history of alcohol or drug dependence, and no use of medication for depression or other mental health disorders.

#### Detailed information on imaging preprocessing

For each subject, the first 10 volumes were discarded to decrease instability factors from MRI acquisition circumstances and subject habituation. Slice timing correction and realignment were further conducted to compensate for head motion artifacts due to breathing, heartbeats, and uncontrolled slight motion during the scan. Eight ScD and seven HC participants were excluded because of excessive head motion (2 mm translation or 2°rotation). MPRAGE structural images were then registered into the Montreal Neurological Institute (MNI) space with a unified segmentation DARTEL algorithm. Nuisance covariates regression with the Friston 24-parameter model (i.e., six head motion parameters, six head motion parameters one timepoint prior, and the 12 corresponding squared items) was also conducted to filter out head motion effects. Next, registration of functional images to the MNI space was performed using structural normalization parameters, and normalization was completed with a resample voxel size of 3 mm x 3 mm x 3 mm. Subsequently, fMRI images were spatially smoothed with full-width at half maximum (FWHM) 6 mm x 6 mm x 6 mm Gaussian kernel. Then, fMRI images were temporally band-pass filtered (0.01-0.08 Hz) to reduce low frequency drift and physiological high frequency respiratory and cardiac noise.
